# Supplementary material for: Relationship Between Level of Trimethylamine Oxide and the Risk of Recurrent Cardiovascular Events in Patients with Acute Myocardial Infarction
Source: Nutrients. 2025 May 14;17(10):1664. doi: 10.3390/nu17101664 (PMC12114086; doi:10.3390/nu17101664)
Supplement: Supplementary file 1 [file nutrients-17-01664-s001.zip › nutrients-3594482-supplementary.pdf]

Supplemental Figure S1. Interactions of risk factors with ln-transformed choline levels and MACE.

Abbreviations: CABG, coronary artery bypass grafting; CI, confidence interval; Cr, creatinine; cTnI, cardiac troponin I; HR, hazard ratio; LDL-C, low-density lipoprotein cholesterol; LVEF, left ventricular ejection fraction; MACE, major adverse cardiovascular events; MI, myocardial infarction; NSTEMI, non-ST-elevation myocardial infarction; PCI, percutaneous coronary intervention; STEMI, ST-elevation myocardial infarction

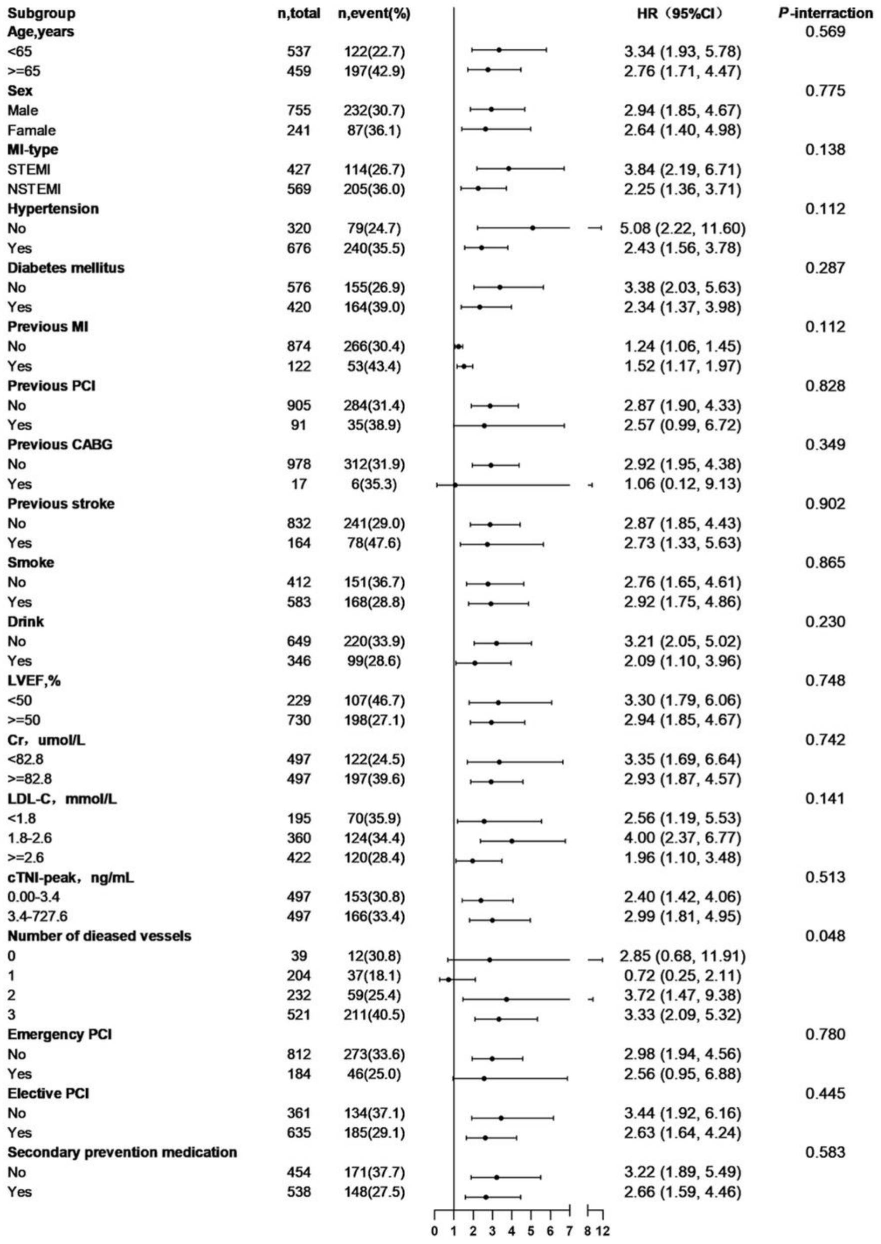

Supplemental Figure S2. Interactions of risk factors with ln-transformed betaine levels and MACE.

Abbreviations: CABG, coronary artery bypass grafting; CI, confidence interval; Cr, creatinine; cTnI, cardiac troponin I; HR, hazard ratio; LDL-C, low-density lipoprotein cholesterol; LVEF, left ventricular ejection fraction; MACE, major adverse cardiovascular events; MI, myocardial infarction; NSTEMI, non-ST-elevation myocardial infarction; PCI, percutaneous coronary intervention; STEMI, ST-elevation myocardial infarction

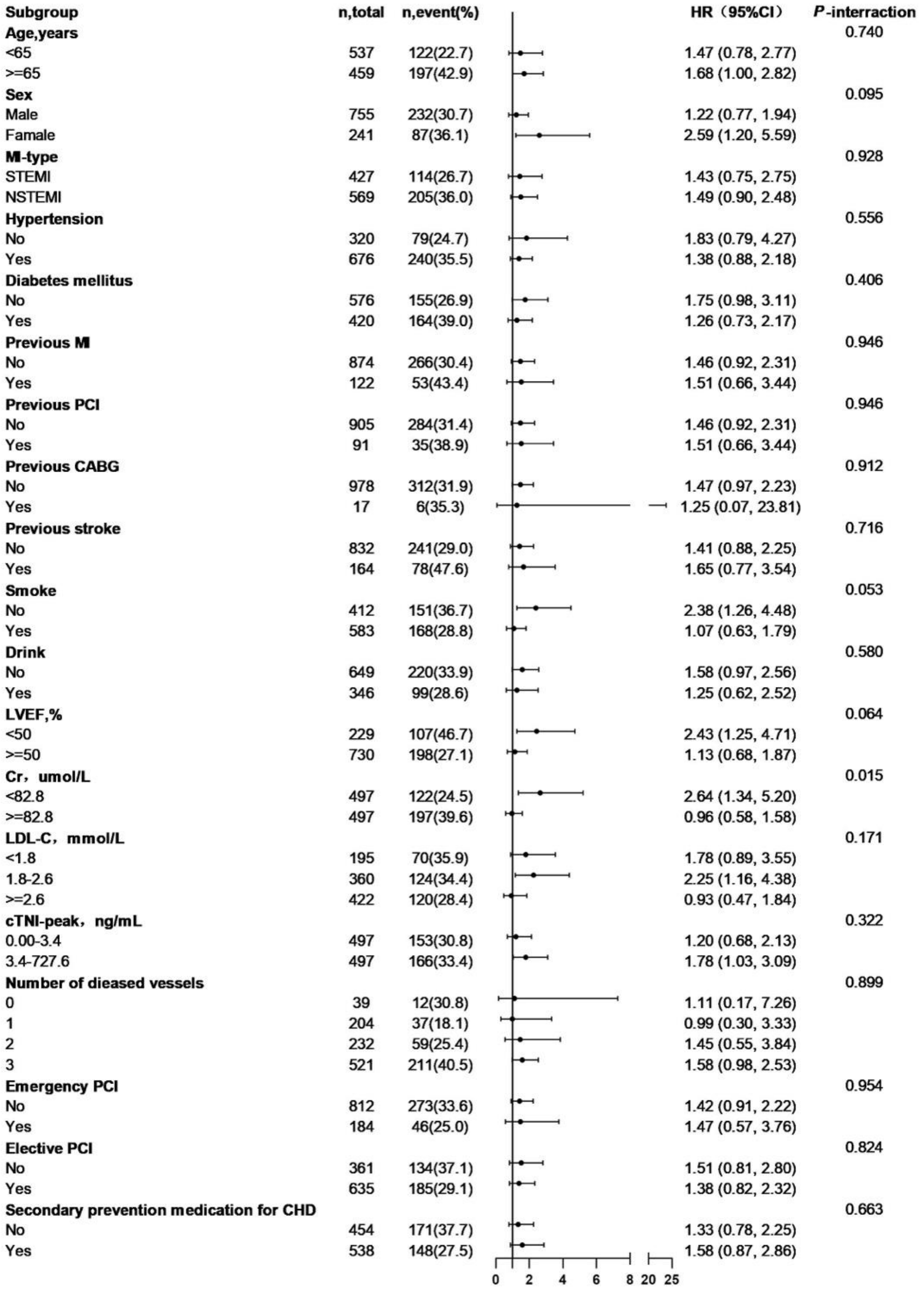

Supplemental Figure S3. Interactions of risk factors with ln-transformed L-carnitine levels and MACE.

Abbreviations: CABG, coronary artery bypass grafting; CI, confidence interval; Cr, creatinine; cTnI, cardiac troponin I; HR, hazard ratio; LDL-C, low-density lipoprotein cholesterol; LVEF, left ventricular ejection fraction; MACE, major adverse cardiovascular events; MI, myocardial infarction; NSTEMI, non-ST-elevation myocardial infarction; PCI, percutaneous coronary intervention; STEMI, ST-elevation myocardial infarction

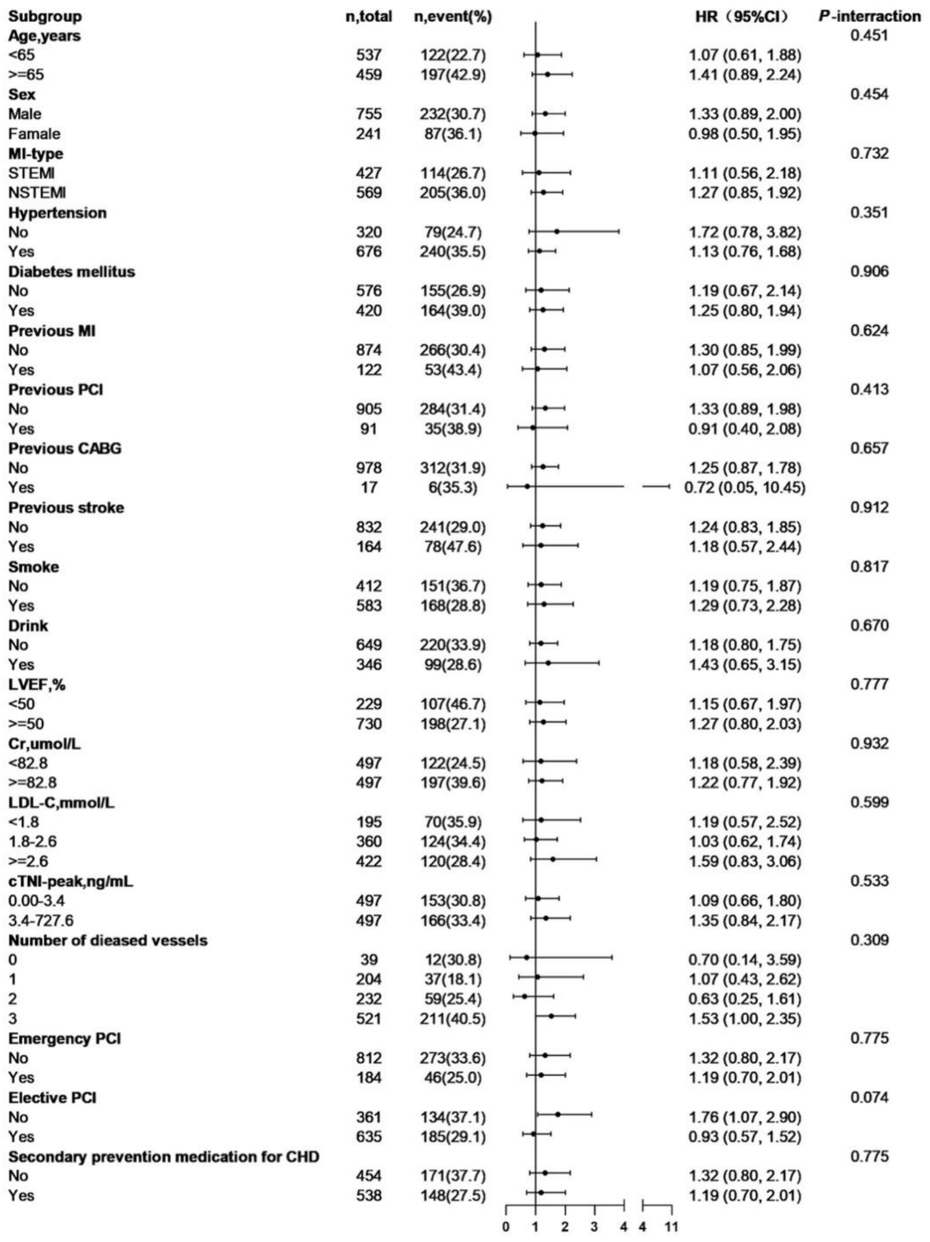

Supplemental Table S1. Source and coding of endpoint events.

endpoint events	source	encodings
cardiovascular death	The Chinese Center for Disease Control and Prevention-National Mortality Surveillance System	I00-I99
myocardial infarction	The Beijing Municipal Health Commission-Beijing inpatient medical record home page systerm	acute ST-segment elevation myocardial infarction (I21.001-006, I21.101-105, I21.201-211, I21.213-230, I21.301, I21.304, I22.001-003, I22.101-103, I22.801-818), acute non-ST-segment elevation myocardial infarction (I21.401-404), other myocardial infarction (I21.302, I21.303, I21.305-308, I21.901, I21.902, I21.907, I21.910, I21.911, I22.901)
	The Chinese Center for Disease Control and Prevention-National Mortality Surveillance System	I21.0, I21.1, I21.2, I21.3, I21.4, I21.9, I22.0, I22.1, I22.8, I22.9
stroke	The Beijing Municipal Health Commission-Beijing inpatient medical record home page systerm	ischemic stroke(I63), hemorrhagic stroke(I60-I61), other strokes(I64)
	The Chinese Center for Disease Control and Prevention-National Mortality Surveillance System	I60, I61, I63, I64

Supplemental Table S2. Associations of the betaine level with all endpoint events.

Model 1 included sex, age, type of MI, history of hypertension, diabetes, myocardial infarction, and stroke, smoking and alcohol consumption, family history of early-onset coronary artery disease, creatinine, triglycerides, high-density lipoprotein cholesterol, low-density lipoprotein cholesterol, cardiac troponin I, left ventricular ejection fraction, number of vessels, emergency PCI for this episode, elective PCI for this episode, discharge medication (aspirin, carbamazepine, statin, beta-receptor antagonist, angiotensin-converting enzyme inhibitor/angiotensin receptor blocker). Model 2 included model 1 plus TMAO, choline, and L-carnitine. *P<0.05. **P<0.001. **Abbreviations:** CI, confidence interval; HR, hazard ratio; MACE, major adverse cardiovascular events; PCI, percutaneous coronary intervention; TMAO, trimethylamine oxide

Endpoint	Group	Event	Crude	Model 1	Model 2
		(n, %)	HR (95%CI)	HR (95%CI)	HR (95%CI)
MACE					
	Ln transform per 1 increase	319 (32.0)	2.06 (1.44, 2.95) **	1.47 (0.98, 2.21)	1.09 (0.69, 1.71)
	Tertile 1	68 (20.5)	1 (Ref)	1 (Ref)	1 (Ref)
	Tertile 2	90 (27.1)	1.17 (0.88, 1.55)	1.09 (0.81, 1.49)	0.98 (0.72, 1.34)
	Tertile 3	161 (48.5)	1.52 (1.16, 1.99) *	1.28 (0.94, 1.73)	1.03 (0.75, 1.43)
	Trend test		0.002	0.108	0.820
All-cause death					
	Ln transform per 1 increase	250 (25.1)	2.06 (1.38, 3.10) **	1.57 (1.00, 2.48) *	1.02 (0.62, 1.68)
	Tertile 1	51 (15.4)	1 (Ref)	1 (Ref)	1 (Ref)
	Tertile 2	66 (19.9)	1.22 (0.89, 1.68)	1.20 (0.85, 1.70)	1.04 (0.73, 1.48)
	Tertile 3	133 (40.1)	1.52 (1.12, 2.07) *	1.43 (1.01, 2.04) *	1.07 (0.74, 1.56)

	Trend test		0.008	0.043	0.718
cardiac death					
	Ln transform per 1 increase	127 (12.8)	3.25 (1.87, 5.67) **	2.59 (1.36, 4.93) *	1.59 (0.77, 3.30)
	Tertile 1	21 (6.3)	1 (Ref)	1 (Ref)	1 (Ref)
	Tertile 2	34 (10.2)	1.08 (0.68, 1.72)	1.08 (0.64, 1.81)	0.87 (0.51, 1.47)
	Tertile 3	72 (21.7)	1.78 (1.16, 2.72) *	1.64 (1.00, 2.68) *	1.13 (0.66, 1.92)
	Trend test		0.006	0.040	0.571
reMI					
	Ln transform per 1 increase	185 (18.6)	1.96 (1.22, 3.14) *	1.77 (1.04, 3.02) *	1.32 (0.74, 2.37)
	Tertile 1	40 (12.0)	1 (Ref)	1 (Ref)	1 (Ref)
	Tertile 2	51 (15.4)	0.76 (0.52, 1.11)	0.73 (0.48, 1.11)	0.65 (0.43, 1.00) *
	Tertile 3	94 (28.3)	1.42 (1.02, 1.99) *	1.38 (0.95, 2.03)	1.11 (0.74, 1.66)
	Trend test		0.032	0.064	0.475
Stroke					
	Ln transform per 1 increase	107 (10.7)	1.57 (0.84, 2.93)	0.74 (0.36, 1.54)	0.60 (0.27, 1.34)
	Tertile 1	21 (6.3)	1 (Ref)	1 (Ref)	1 (Ref)
	Tertile 2	31 (9.3)	1.36 (0.85, 2.18)	1.01 (0.61, 1.67)	0.97 (0.58, 1.61)
	Tertile 3	55 (16.6)	1.30 (0.80, 2.11)	0.82 (0.47, 1.40)	0.72 (0.40, 1.28)
	Trend test		0.292	0.440	0.255

Supplemental Table S3. Associations of the L-carnitine level with all endpoints

Model 1 included sex, age, type of MI, history of hypertension, diabetes, myocardial infarction, and stroke, smoking and alcohol consumption, family history of early-onset coronary artery disease, creatinine, triglycerides, high-density lipoprotein cholesterol, low-density lipoprotein cholesterol, cardiac troponin I, left ventricular ejection fraction, number of vessels, emergency PCI for this episode, elective PCI for this episode, discharge medication (aspirin, carbamazepine, statin, beta-receptor antagonist, angiotensin-converting enzyme inhibitor/angiotensin receptor blocker). Model 2 included model 1 plus TMAO, choline, and betaine. **P*<0.05. **Abbreviations:** CI, confidence interval; HR, hazard ratio; MACE, major adverse cardiovascular events; PCI, percutaneous coronary intervention; TMAO, trimethylamine oxide

Endpoint	Group	Event (n, %)	Crude HR (95%CI)	Model 1 HR (95%CI)	Model 2 HR (95%CI)
MACE					
	Ln transform per 1 increase	319 (32.0)	1.44 (0.98, 2.13)	1.23 (0.86, 1.75)	1.02 (0.71, 1.48)
	Tertile 1	68 (20.5)	1 (Ref)	1 (Ref)	1 (Ref)
	Tertile 2	90 (27.1)	1.06 (0.81, 1.40)	1.15 (0.86, 1.54)	1.08 (0.80, 1.45)
	Tertile 3	161 (48.5)	1.15 (0.88, 1.51)	1.17 (0.88, 1.57)	1.05 (0.79, 1.41)
	Trend test		0.296	0.286	0.740
All-cause death					
	Ln transform per 1 increase	250 (25.1)	1.61 (1.03, 2.50) *	1.25 (0.86, 1.82)	0.95 (0.64, 1.41)
	Tertile 1	51 (15.4)	1 (Ref)	1 (Ref)	1 (Ref)
	Tertile 2	66 (19.9)	0.92 (0.67, 1.26)	1.07 (0.76, 1.51)	0.97 (0.69, 1.37)
	Tertile 3	133 (40.1)	1.08 (0.80, 1.46)	1.05 (0.75, 1.45)	0.90 (0.64, 1.25)

	Trend test		0.601	0.799	0.514
cardiac death					
	Ln transform per 1 increase	127 (12.8)	2.36 (1.32, 4.20) *	1.78 (1.07, 2.96) *	1.33 (0.76, 2.31)
	Tertile 1	21 (6.3)	1 (Ref)	1 (Ref)	1 (Ref)
	Tertile 2	34 (10.2)	1.03 (0.66, 1.61)	1.13 (0.69, 1.85)	0.97 (0.59, 1.61)
	Tertile 3	72 (21.7)	1.27 (0.83, 1.93)	1.25 (0.79, 2.00)	1.03 (0.64, 1.66)
	Trend test		0.266	0.339	0.909
reMI					
	Ln transform per 1 increase	185 (18.6)	1.01 (0.60, 1.69)	0.92 (0.57, 1.46)	0.87 (0.54, 1.42)
	Tertile 1	40 (12.0)	1 (Ref)	1 (Ref)	1 (Ref)
	Tertile 2	51 (15.4)	1.02 (0.72, 1.46)	1.04 (0.71, 1.53)	1.01 (0.68, 1.49)
	Tertile 3	94 (28.3)	1.09 (0.77, 1.55)	1.11 (0.76, 1.61)	1.06 (0.72, 1.54)
	Trend test		0.621	0.589	0.780
Stroke					
	Ln transform per 1 increase	107 (10.7)	1.02 (0.60, 1.74)	1.09 (0.68, 1.75)	0.92 (0.56, 1.51)
	Tertile 1	21 (6.3)	1 (Ref)	1 (Ref)	1 (Ref)
	Tertile 2	31 (9.3)	1.07 (0.74, 1.52)	1.12 (0.76, 1.65)	1.04 (0.70, 1.53)
	Tertile 3	55 (16.6)	1.09 (0.76, 1.55)	1.31 (0.90, 1.91)	1.17 (0.79, 1.71)
	Trend test		0.643	0.165	0.434
